# Optimized Saturation Pulse Train for Human First-Pass Myocardial Perfusion Imaging at 7T

**DOI:** 10.1002/mrm.25262

**Published:** 2014-04-18

**Authors:** Yuehui Tao, Aaron T Hess, Graeme A Keith, Christopher T Rodgers, Alexander Liu, Jane M Francis, Stefan Neubauer, Matthew D Robson

**Affiliations:** Radcliffe Department of Medicine, University of Oxford, Oxford Centre for Clinical Magnetic Resonance Research (OCMR), John Radcliffe HospitalOxford, OX3 9DU, UK.

**Keywords:** 7T, cardiac magnetic resonance imaging, myocardial perfusion, saturation, radiofrequency pulse, B_1_ inhomogeneity

## Abstract

**Purpose:**

To investigate whether saturation using existing methods developed for 3T imaging is feasible for clinical perfusion imaging at 7T, and to propose a new design of saturation pulse train for first-pass myocardial perfusion imaging at 7T.

**Methods:**

The new design of saturation pulse train consists of four hyperbolic-secant (HS8) radiofrequency pulses, whose peak amplitudes are optimized for a target range of static and transmit field variations and radiofrequency power deposition restrictions measured in the myocardium at 7T. The proposed method and existing methods were compared in simulation, phantom, and in vivo experiments.

**Results:**

In healthy volunteer experiments without contrast agent, average saturation efficiency with the proposed method was 97.8%. This is superior to results from the three previously published methods at 86/95/90.8%. The first series of human first-pass myocardial perfusion images at 7T have been successfully acquired with the proposed method.

**Conclusion:**

Existing saturation methods developed for 3T imaging are not optimal for perfusion imaging at 7T. The proposed new design of saturation pulse train can saturate effectively, and with this method first-pass myocardial perfusion imaging is feasible in humans at 7T. **Magn Reson Med 73:1450–1456, 2015.** © 2014 The Authors. Magnetic Resonance in Medicine Published by Wiley Periodicals, Inc. on behalf of International Society of Medicine in Resonance.

## INTRODUCTION

First-pass myocardial perfusion imaging (1) is one of the most important applications of cardiac magnetic resonance (MR). It has been reported that 3T cardiac MR perfusion imaging is superior to 1.5T for prediction of significant single vessel and multivessel coronary disease (2). Further improvement can be expected at 7T due to higher signal-to-noise ratio and longer T_1_ of the myocardium (3) but no clinical cardiac perfusion at 7T has yet been reported.

The major challenges at 7T are large static field (B_0_) and transmit radiofrequency (RF) field (B_1_) variations across the heart and limited RF power deposition allowed for a complete and uniform saturation. Incomplete saturation in the perfusion sequence can result in unintended high signal in the myocardium in perfusion images, which may make it difficult to distinguish a perfusion defect from healthy myocardium. This problem was already observed when transitioning from 1.5T to 3T. Perfusion methods developed at 1.5T were found to operate inadequately at 3T owing to large B_0_ and B_1_ variation due to limited capability of commercially available shimming and calibration methods for the heart. Several methods have been proposed to overcome these challenges. Composite adiabatic pulses known as B_1_-insensitive rotation (BIR4) (4,5) have been demonstrated capable of effective saturation at 3T (6). A train of two BIR4 pulses has also been proposed for saturation at 3T (7,8). The disadvantage of the BIR4 pulse is that it has an excessive specific absorption rate (SAR), which is problematic even at 3T (6).

Another group of saturation methods use a train of RF pulses. Each RF subpulse is interleaved between gradient spoilers to remove residual transverse magnetization. Perhaps the simplest variation uses a pulse train of three nonselective 90° rectangular pulses (9) (hereafter referred to as the *standard* pulse train). This method is adopted on commercially available 1.5T and 3T systems but has been shown to be susceptible to B_1_ inhomogeneity at 3T (8,10). Alternatively, the *tailored* pulse train (10) consists of rectangular pulses of different flip angles. The flip angles are optimized for a range of B_0_ and B_1_ variation measured a priori over the heart. The *hybrid* pulse train (8) is an extension of the *tailored* pulse train, combining two rectangular pulses and an adiabatic half-passage pulse. Both the *tailored* and *hybrid* pulse trains produced better saturation than the *standard* pulse train at 3T (8,10), but their performance depends on the predetermined range of B_0_ and B_1_ variation that the pulse trains were optimized for. The B_1_-insensitive train to obliterate signal BISTRO (11), developed for water suppression and outer-volume suppression when using local transmit coils, uses a large number of amplitude- and frequency-modulated RF pulses, each of which is offset-independent adiabatic and produces a uniform saturation inside the limits of a frequency sweep range. To compensate for large B_1_ variation, the amplitudes of the RF pulses in the BISTRO pulse train are varied. A sufficiently large number of RF pulses are required to ensure that all regions experience a saturating flip angle at least once. The limitation of the BISTRO method is that the RF power must exceed a threshold level and the required number of RF pulses can be large [32 in (11)].

According to initial experience with our 7T whole body system, the range of B_0_ and B_1_ variation is much larger than at 3T, and the peak B_1_ level that is available is much lower. Further, the SAR limit becomes much more challenging at 7T owing to the higher frequency of the RF. Consequently, uniform saturation presents many challenges.

In this study, we first determine the range of B_0_ and B_1_ and the effect of the SAR limit from scans of healthy volunteers on our 7T system. Then, we investigate whether saturation using existing methods developed at 3T is feasible for perfusion imaging at 7T. Further, we propose a new design of saturation pulse train for first-pass myocardial perfusion imaging at 7T. The new method is compared to existing techniques in simulation, phantom, and in vivo experiments. We also present the first series of human first-pass myocardial perfusion images at 7T.

## METHODS

All experiments used a Magnetom whole-body 7T MRI scanner (Siemens Healthcare, Erlangen, Germany). The coil is an eight-channel strip-line transverse electromagnetic transceiver array (12). Maximum gradient strength is 70 mT/m, and maximum slew rate is 200 T/m/s.

A fast low-angle shot sequence was modified to be able to use different saturation pulse trains prior to image acquisition. All images were acquired in the midventricular short-axis slice. One image was collected in each heartbeat with the perfusion sequence. The Siemens vector electrocardiogram was used for triggering, with a trigger delay of 300 ms. Unless otherwise stated, the imaging parameters were: field of view 380 × 285 mm^2^; acquisition matrix 192 × 144; slice thickness 8 mm; in-plane resolution 2 × 2 mm^2^; nominal flip angle 10°; echo time 1.18 ms; repetition time 2.82 ms; receiver bandwidth 1002 Hz/pixel. Generalized autocalibrating partially parallel acquisitions (13) was applied with an acceleration factor of 2. The acquisition of each image consisted of 84 phase-encoding steps. Time between the end of the saturation pulse train and the central k-space readout was 125 ms.

Eight healthy volunteers (age 31 ± 7 years, weight 77 ± 8 kg) were recruited. Ethics approval was granted for all study procedures and informed consent was obtained from all subjects.

### B_0_ and B_1_ Variation

Manufacturer provided software was used for B_0_ shimming over an adjustment volume covering the whole left ventricle (LV). B_0_ offset was measured with a double-echo gradient echo sequence.

Local B_1_ shimming (14) was performed to maximize the minimum B_1_ over a region of interest (ROI) manually selected in the midventricle short-axis slice covering the LV. The B_1_ map was determined from images acquired with a B_0_-independent saturation preparation method (15).

B_0_ and B_1_ maps in the midventricle short-axis slice were acquired from eight volunteers. LV myocardium was manually segmented in the B_0_ and B_1_ maps. The observed number of pixels as a function of B_0_ and B_1_ was used to fit the joint probability distribution for B_0_ and B_1_ in the LV myocardium. The distribution was approximated as a Gaussian mixture distribution (16) in the fitting as in the design of the *tailored* pulse train (10). A range of B_0_ offset (−250 to 250 Hz) and B_1_ (150–400 Hz, 1 µT of B_1_ corresponds to 42.58 Hz) was then manually selected to cover the aforementioned distribution with a probability greater than 99%.

### Energy Limit for the Saturation Pulse Train

The online RF-power monitoring system of the scanner was used to determine the maximum energy available for the saturation pulse train. The system monitors RF power averaged in real-time over durations of 0.1/1/10/360 s, and compares it with a set of preset RF power limits. A scan will be stopped automatically when any of the preset RF power limits is exceeded. Each transmit element of the eight-channel coil is connected to an independently controlled RF amplifier and dedicated power monitor. Conservative safety limits are imposed based on SAR simulations for the coil in Semcad X (Schmid and Partner Engineering AG, Switzerland). These worst-case limits assume constructive interference of E-fields everywhere in space. The preset safety limit that is most likely breached is the 1 s limit. For perfusion scans saturating once and collecting one image per heartbeat, the total energy of all RF pulses within 1 s also depends on the heart rate. For a typical heart rate between 50 and 100 bpm, the design of the saturation pulse train needs to allow both saturation and image acquisition to be applied twice within 1 s.

The limit of relative energy for a saturation pulse train was obtained using a maximum-amplitude rectangular pulse as the saturation pulse for the perfusion pulse sequence and gradually increasing the duration of the rectangular pulse, in steps of 0.1 ms, to challenge the RF-power monitoring system. The relative energy is defined as the total energy of a set of RF pulses divided by the energy of a 1-ms rectangular pulse of the maximum amplitude.

According to the limits measured from eight volunteers and assuming a normal distribution, the limit of relative energy for a saturation pulse train was set to 6. With the limit at this level, the probability of triggering an automatic stop of scan due to excessive energy deposition by the saturation RF pulses is less than 1% at a heart rate between 50 and 100 bpm. This limit would be breached if an 8-ms BIR4 pulse at maximum amplitude was used for saturation.

### Optimization of Saturation Pulse Train for 7T

The development of the saturation pulse train for perfusion at 7T used the same approach adopted for the *tailored* pulse train (10). The flip angles (peak amplitudes) of the RF pulses in the pulse train were optimized over the range of B_0_ and B_1_ variation determined above. Large B_0_ variation requires RF pulses of broad bandwidth (hence a short duration high voltage pulse), while regions of low peak B_1_ also increase the demands on the coil voltage. It is difficult to fulfill both requirements with a rectangular pulse of fixed amplitude because its flip angle increases with its duration, but its bandwidth decreases proportionally. No good solution was found in the search for a tailored rectangular pulse train under the conditions at 7T.

Full-passage hyperbolic-secant (HS8) pulses (17) are more suited to this saturation problem. When the duration of a HS8 pulse is fixed at 5 ms, and the frequency sweep ±500 Hz, the effective bandwidth is not sensitive to the change of peak amplitude at low levels of peak B_1_ comparable to the B_1_ range at 7T.

To minimize the relative energy [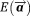] of the HS8 pulse train, an exhaustive search of the peak amplitudes [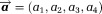] of the four HS8 pulses (duration 5 ms, frequency sweep ±500 Hz) was carried out. The peak amplitudes ranged from 0 to 100% of the maximum amplitude, in steps of 1%. Numerical Bloch simulations were performed over the range (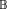) of B_0_ offset (±250 Hz) and B_1_ (150–400 Hz) previously determined from volunteer measurements. T_1_ and T_2_ relaxation was ignored. Ideal spoiling was assumed in the simulation, so the transverse magnetization was zero before each RF pulse. Solutions were excluded when the normalized residual longitudinal magnetization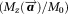 was outside the range −6% to 0, or when the relative energy of the four HS8 pulses was greater than 6.


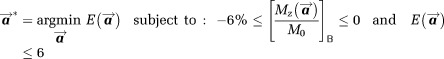


**FIG 1:**
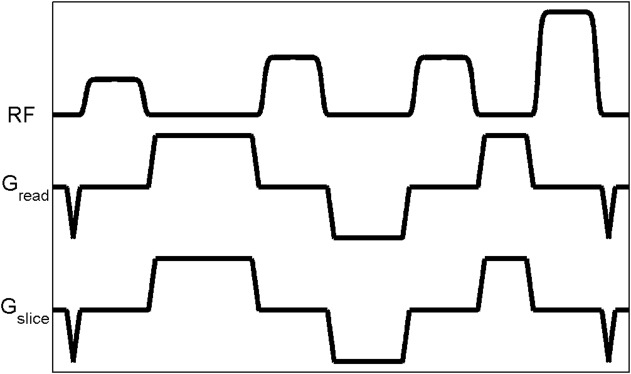
Pulse sequence diagram of the HS8 saturation pulse train.

The peak amplitudes determined from the search are 29/47/47/84%, respectively, of the maximum amplitude. The relative energy of the HS8 pulse train is 4.9. The probability of a stop of scan triggered by the HS8 pulse train due to excessive RF energy is virtually zero, according to the distribution of the energy limits previously measured from eight volunteers. To complete the HS8 pulse train, each HS8 pulse is interleaved between gradient spoilers (duration 1/8/6/4/1 ms including 0.5 ms for each linear ramp, peak amplitude 50 mT/m), as shown in Figure 1. The durations and amplitudes of the gradient spoilers were determined empirically to ensure adequate spoiling.

### Simulation, Phantom, and In Vivo Comparisons

The saturation performance of the HS8 pulse train and four existing methods (the *standard*/*tailored*/*hybrid* pulse train and the BIR4 pulse) was evaluated in numerical Bloch simulation of the saturation of a single isochromat in the B_1_ range of 150–400 Hz and B_0_ offset range of ±250 Hz. The parameters of the five methods are listed in Table 1. T_1_ and T_2_ relaxation was ignored and ideal spoiling was assumed.

**Table 1:** Parameters of the Saturation Pulse Trains

	Pulse train		RF pulse	
	
	Relative energy^a^	Duration^b^ (ms)	Duration (ms)	Peak amplitude^c^
Standard (9,10)	1.5	6.5	0.5	1
			0.5	1
			0.5	1
BIR4 (4,10)	6.4	8	8 (BIR4)	1
Tailored (10)	2.648	7.648	0.548	1
			1.296	1
			0.804	1
Hybrid (8)	2.1	7.84	0.8	0.73
			0.5	0.75
			1.54 (AHP)	1
HS8 (17)	4.9	38	5 (HS8)	0.29
			5 (HS8)	0.47
			5 (HS8)	0.47
			5 (HS8)	0.84

a Relative energy is defined as the total energy of the pulse train divided by the energy of a 1-ms rectangular RF pulse at the maximum amplitude.

b Pulse train duration excludes leading and trailing gradient spoilers. The spoiler durations for the standard/tailored/hybrid pulse trains are 1, 3, 2, and 3 ms. The spoiler durations for the HS8 pulse train are 1, 8, 6, 4, and 1 ms, including 0.5 ms for each linear ramp.

c Peak amplitude is relative to the maximum amplitude.

The HS8 pulse train and three existing methods (the *standard*/*tailored*/*hybrid* pulse train) were also tested in scans of a 10-cm spherical oil phantom (T_1_ 1420 ms). To simulate the B_0_ and B_1_ variation, the amplitude of the saturation RF pulses were scaled from 20 to 100% of the maximum, in steps of 10% of the maximum amplitude, and a frequency offset between ±320 Hz, in steps of 20 Hz, was added to all saturation RF pulses. All phantom images were acquired in the same axial plane at isocenter. In each scan, the test saturated image was collected 8 s after an unsaturated image of otherwise identical imaging parameters.

Each test saturated image was divided pixel-wise by its corresponding unsaturated image to calculate the normalized absolute residual longitudinal magnetization, which was then corrected for T_1_ relaxation effects. The correction calculated the normalized residual longitudinal magnetization at the end of the saturation pulse train from signal measured 125 ms after, assuming simple T_1_ relaxation (T_1_ of oil 1420 ms). Six 2 × 2 (4 pixels) ROIs were selected at locations of different B_1_ levels in each image. The normalized absolute residual longitudinal magnetization was then averaged in each ROI. The B_1_ field over the imaged slice was not homogeneous, so the six ROIs in each image could be selected at locations of desired B_1_ levels. With the scaling of the amplitudes of the saturation RF pulses, 26 B_1_ levels, in steps of 10 Hz, in the range of 150–400 Hz were measured.

Eight volunteers were scanned without contrast using the HS8 pulse train and three existing methods (the *standard*/*tailored*/*hybrid* pulse train). As in the phantom scans, two short axis images were acquired in each breath hold, the first unsaturated and the second saturated. The delay between the two images was 8 s. The LV myocardium was manually segmented into six segments (18). Each segment was treated as an independent ROI. The residual signal was normalized by calculating a pixel-wise ratio image between each saturated image and its corresponding unsaturated image. Mean and maximum values of the normalized residual signal were calculated over each ROI. Mean ± (standard deviation) of the mean and maximum values were then calculated over all 48 ROIs.

### Rest Perfusion Scan

Three male volunteers were scanned during the first pass of gadolinium-chelate contrast agent (Dotarem, Guerbet, France). The HS8 pulse train was used for saturation. 60 images were collected, one each heartbeat, over an end-expiratory breath hold. The volunteers were asked to hold breath as long as possible, and then breathe smoothly. The peripheral injection of a contrast bolus (0.05 mmol/kg, flow rate 6 mL/s) with a power injector (Accutron MR, MEDTRON, Germany) was started between the third and fourth heartbeat. The first image was not saturated.

The LV myocardium was manually segmented into six segments. The signal intensities were averaged in each segment. The average intensities from all images except the first one were divided by the corresponding averages from the first image to calculate the normalized signal intensity. The normalized signal intensity was also calculated for the LV blood pool.

## RESULTS

**FIG 2:**
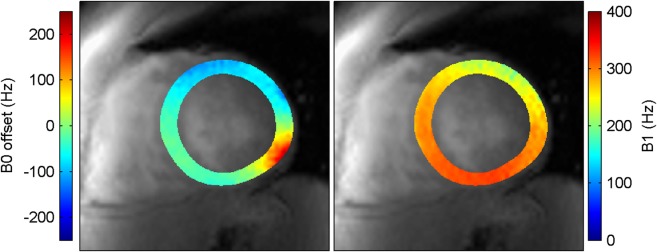
B_0_ offset and B_1_ maps over the LV myocardium measured from one volunteer in the midventricular short-axis slice.

**FIG 3:**
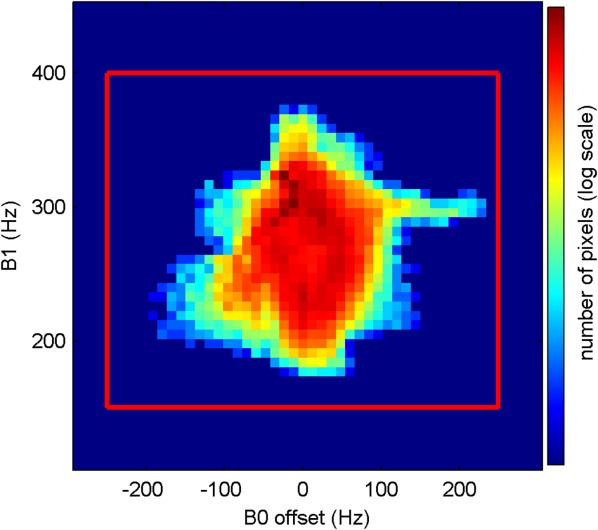
Histogram of B_0_ offset and B_1_ measurements over the LV myocardium from eight volunteers.

Figure 2 shows an example of B_0_ and B_1_ variation from the same volunteer. In the inferolateral segment the B_0_ variation is visibly larger than other regions. This is also observed in images from all other volunteers and is consistent with the literature (19). Figure 3 shows the B_0_ and B_1_ variations measured from all volunteers with the range selected for the optimization of the saturation pulse train.

**FIG 4:**
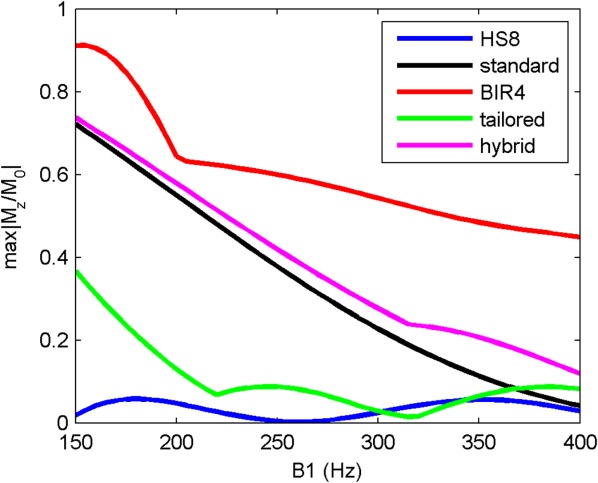
The maximum (worst case) absolute normalized residual longitudinal magnetization (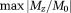) over the B_0_ offset range of ±250 Hz as a function of B_1_, from numerical Bloch simulations.

**FIG 5:**
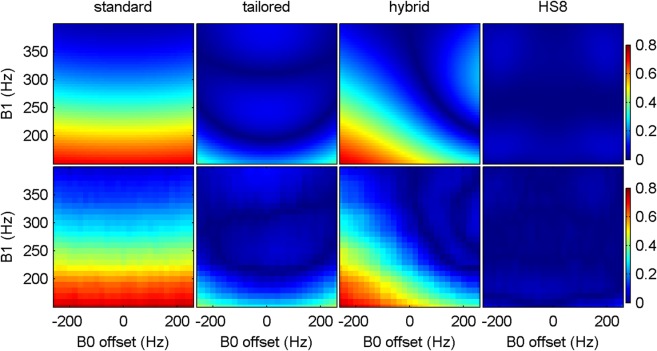
The absolute normalized residual longitudinal magnetization produced with the standard, tailored, hybrid, and HS8 pulse trains in simulation (top row) and phantom scans (bottom row).

Figure 4 shows the worst case results obtained with different methods in simulation. All methods except the HS8 pulse train left residual magnetization over 10% when B_1_ was below 200 Hz. This is also shown in Figure 5 with results from phantom experiments, which agree well with the simulation results. The effective bandwidth of the *tailored* train is broader than that of the *hybrid* train, leading to a lower maximum absolute normalized residual longitudinal magnetization than that from the *hybrid* train over the whole range of B_1_ from 150 to 400 Hz.

**FIG 6:**
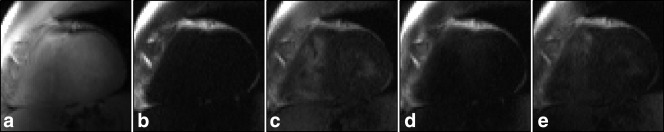
Representative images from one volunteer: (a) nonsaturated, saturated with the (b) HS8, (c) standard, (d) tailored, (e) hybrid pulse train. All saturated images (b–e) are displayed in the same grayscale.

Figure 6 shows the images saturated with different methods, and statistics of mean and maximum residual signal intensity over the LV myocardium from eight volunteers are listed in Table 2. Given that the relaxation time between the end of saturation pulse train and the central k-space readout was 125 ms, the normalized residual myocardium signal immediately after the saturation was calculated assuming T_1_ in the myocardium was 1950 ms (3). The average residual from the HS8 train was 2.2% (average saturation efficiency 97.8%), less than the average residual from the *standard*/*tailored*/*hybrid* trains at 14.0/5.0/9.2% (average saturation efficiency 86/95/90.8%). The maximum residual from the HS8 train was 4.7%, less than the maximum residual from the *standard*/*tailored*/*hybrid* trains at 25.2/13.3/19.2%.

**Table 2:** Mean (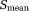) and Maximum (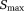) Residual Signal Intensity Over the Left Ventricle Myocardium Measured from All Volunteers before T_1_ Correction

Saturation pulse train	*S*_mean_ (%)	*S*_max_ (%)
HS8	8.3 ± 0.9	10.6 ± 1.1
Standard	19.3 ± 7.1	29.8 ± 12.5
Tailored	10.9 ± 4.2	18.7 ± 6.5
Hybrid	14.8 ± 2.2	24.2 ± 7.1

**FIG 7:**
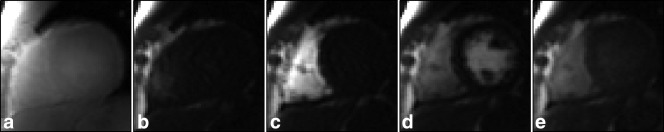
First-pass perfusion images with the HS8 pulse train for saturation: (a) nonsaturated, (b) before arrival of contrast agent, (c) right ventricle blood enhancement, (d) LV blood enhancement, (e) LV wall enhancement. All saturated images (b–e) are displayed in the same grayscale.

**FIG 8:**
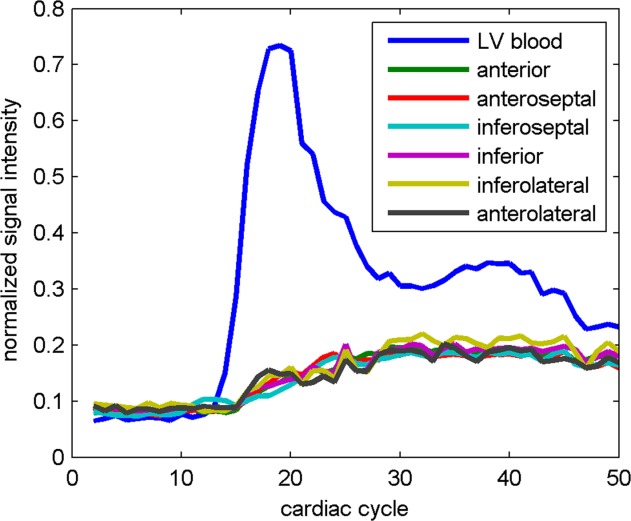
The time course of the normalized signal intensity in LV blood pool and six midventricular short-axis segments (18) during the first pass of the contrast agent.

Figures 7 and 8 shows first-pass perfusion results from a male volunteer (age 32 years, weight 80 kg). The normalized residual signals from all segments are close to each other, suggesting a uniform saturation in this healthy volunteer. These images demonstrate that first-pass perfusion imaging in humans at 7T is feasible.

## DISCUSSION

With this work, we have developed a new design of saturation pulse train for cardiac perfusion imaging at 7T, and successfully acquired the first series of human first-pass myocardial perfusion images at 7T with the proposed method. In simulation, phantom, and in vivo experiments, we have demonstrated that existing saturation methods developed for 3T imaging are not optimal for cardiac perfusion imaging at 7T. This can be explained by differences in the ranges of B_0_ and B_1_ variation targeted by these saturation methods. Previously proposed methods were designed for a higher B_1_ level and narrower B_0_ bandwidth. These were appropriate for body coil on 3T scanners but not suitable for the transmitting surface coil on our 7T system. Lower B_1_ levels require more power for the saturation, however, the RF power deposition allowed at 7T is very limited. This excluded the BIR4 pulse, and limited the number of pulses available for use in all pulse trains. The HS8 pulse and the adiabatic half-passage pulse in the *hybrid* pulse train are both operating outside adiabatic range at low B_1_ levels in the targeted range of B_1_ at 7T, which may explain the relatively high residual normalized signal after the saturation with the *hybrid* pulse train.

The range of B_0_ offset (±250 Hz) and B_1_ (150–400 Hz) determined from in vivo measurements had an important role in the design of the HS8 pulse train. No good solution was found in the search for a tailored rectangular pulse train for this range. The duration and frequency sweep of the HS8 pulses were specifically chosen for the above range. The results of the search were sensitive to changes to the range, especially changes to the lower limit of the B_1_ range. With the lower B_1_ limit at 140 Hz, no solution was found. At 200 Hz, the required relative energy was reduced to 2.7. Above 235 Hz, the required relative energy was less than 2, enabling coverage of three slices. Reducing the higher limit of the B_1_ range did not save much energy. With the higher limit at 320 Hz, the saturation still required 4.8 in relative energy. Because the effective saturation bandwidth of the chosen HS8 pulse is mainly determined by its duration and frequency sweep, the results of the search were not sensitive to reduction of the B_0_ offset bandwidth. With the bandwidth reduced to ±150 Hz, the required relative energy was 4.8. If the range was redefined as an elliptical region spanning ±250 Hz in B_0_ offset and 150–400 Hz in B_1_, the required relative energy would be 4.4. The HS8 pulse train proposed in this work (29/47/47/84%) would not saturate effectively if B_0_ offset and B_1_ were out of the predetermined range. According to numerical Bloch simulation ignoring relaxation, the maximum normalized longitudinal magnetization immediately after saturation would be 5.6% at B_1_ of 135 Hz and 0.2% at 415 Hz. If the B_0_ offset bandwidth was ±300 Hz, the maximum normalized longitudinal magnetization would be 5.4%. Within the range selected in this work, the performance of the HS8 pulse train is not sensitive to small deviations in amplitude of any single HS8 pulse in the pulse train. If the peak amplitude of any single RF pulse in the HS8 pulse train changes by 1% of the maximum amplitude, the maximum change in minimum or maximum normalized longitudinal magnetization will be less than 0.01.

The HS8 pulse train bears an appearance similar to the BISTRO method, both using amplitude- and frequency-modulated RF pulses interleaved between gradient spoilers. The RF pulses used in the BISTRO method have a wide frequency sweep (8 KHz), and they are offset-independent adiabatic. The frequency sweep of the HS8 pulses in the HS8 pulse train in this work is only 1000 Hz. In the low B_1_ field observed in this study, these 5-ms HS8 pulses are not offset-independent adiabatic. The peak amplitudes of the RF pulses of the BISTRO method are not optimized for a target range of B_0_ and B_1_ variation, and the BISTRO method requires a sufficiently large number of RF pulses to ensure that all regions experience a flip angle near 90° at least once, which was not required in the design of the HS8 pulse train. In regions of low B_1_, each single HS8 pulse alone in the HS8 pulse train will not produce a flip angle of 90°, but the combined effect of four HS8 pulses will produce a saturating flip angle.

Other types of numerically optimized RF pulses can also be used to build the saturation pulse train. However, it is difficult to cover the whole universe of RF pulses. We did evaluate a selection of different pulses and found HS8 to be the best. In addition to a pulse train of all HS8 pulses, we also observed in numerical Bloch simulations that a hybrid train of rectangular and HS8 pulses could also saturate effectively in the target B_0_ and B_1_ range but that it deposits slightly greater power into the subject. At low flip angles (short pulse durations), a hard pulse may have similar saturation effects as an HS8 pulse of slightly less power, when the effective bandwidth of the hard pulse is wide enough to cover the target range of B_0_. An optimized hybrid train of HS8 and hard pulses may be built by replacing low-flip-angle HS8 pulses in the optimized HS8 pulse train with hard pulses of slightly greater power. However, the high-flip-angle HS8 pulses cannot be replaced in the same way, because hard pulses of high flip angles (long durations) will not saturate effectively over the target range of B_0_. This may explain why no good solution was found in the search for a tailored rectangular pulse train under the conditions at 7T.

It should be noted that the imaging protocol used for the rest perfusion scan was not optimized specifically for 7T. The image quality in these first acquisitions is already comparable to that at 3T, supporting the great potential for perfusion imaging at 7T.

Multislice cardiac perfusion imaging at 7T which saturates multiple times in each heart beat remains challenging. According to the relative energy limit allowed for the saturation pulse measured in this study, the relative energy of the saturation pulse train needs to be reduced to 2 if the heart is to be saturated three times each heartbeat. One option may be to improve the B_1_ shimming performance. If the lower limit of B_1_ can be increased to 200 Hz and the upper limit unchanged, the RF energy of the saturation pulse train may be reduced by 43% by simply scaling down the amplitudes of the RF pulses. Alternatively improved B_0_ shimming would allow the optimization to be performed over a narrower B_0_ range which would be a second means of reducing RF power deposition. Finally, the RF SAR calculations that we have used are by necessity cautious and future improvements in RF coils and/or RF monitoring technology should allow us to deliver more power into the subject in complete safety. These are all areas of on-going development.

## CONCLUSIONS

We demonstrated that the proposed HS8 pulse train saturated effectively within the target range of B_0_ and B_1_ variation and the energy limit at 7T, while the existing methods designed for 3T imaging were not as effective. We also demonstrated that single-slice first-pass myocardial perfusion imaging is feasible for human at 7T.
